# Patient-Reported Outcome of Physical Therapy in Amyotrophic Lateral Sclerosis: Observational Online Study

**DOI:** 10.2196/10099

**Published:** 2018-11-12

**Authors:** Robert Meyer, Susanne Spittel, Laura Steinfurth, Andreas Funke, Dagmar Kettemann, Christoph Münch, Thomas Meyer, André Maier

**Affiliations:** 1 Center for ALS and other Motor Neuron Disorders Department of Neurology Charité – Universitätsmedizin Berlin, corporate member of Freie Universität Berlin, Humboldt-Universität zu Berlin, and Berlin Institute of Health Berlin Germany; 2 Ambulanzpartner Soziotechnologie APST GmbH Berlin Germany

**Keywords:** ALS, amyotrophic lateral sclerosis, physical therapy, MYMOP, net promoter score, NPS, online self-assessment

## Abstract

**Background:**

Physical therapy is an essential component of multidisciplinary treatment in amyotrophic lateral sclerosis (ALS). However, the meaning of physical therapy beside preservation of muscular strength and functional maintenance is not fully understood.

**Objective:**

The purpose of this study was to examine patients’ perception of physical therapy during symptom progression using an internet assessment approach.

**Methods:**

A prospective, longitudinal, observational study was performed. Recruitment took place in an ALS center in Berlin, Germany. Online self-assessment was established on a case management platform over 6 months. Participants self-assessed the progression of the disease with the ALS Functional Rating Scale-Revised (ALSFRS-R) and tracked the efficacy of targeted physical therapy using Measure Yourself Medical Outcome Profile (MYMOP). We used the net promoter score (NPS) to inquire into recommendation levels of physical therapy.

**Results:**

Forty-five participants with ALS were included in the study. Twenty-seven (60.0%) started the online assessment. The mean duration of physical therapy sessions per week was 142.7 minutes (SD 60.4) with a mean frequency of 2.9 (SD 1.2) per week. As defined by MYMOP input, the most concerning symptoms were reported in the legs (62.2%), arms (31.1%), and less frequently in the torso (6.7%). As expected for a progressive disease, there was a functional decline of 3 points in the ALSFRS-R at the end of the observation period (n=20). Furthermore, the MYMOP showed a significant loss of 0.8 in the composite score, 0.9 in the activity score and 0.8 in the targeted symptom. In spite of functional decline, the recommendation for physical therapy jumped from a baseline value of 20 NPS points to a very high 50 points at the end of study (*P*=.05).

**Conclusions:**

Physical therapy is perceived as an important treatment method by patients with ALS. Despite functional deterioration, patients are satisfied with physical therapy and recommend this intervention. The results also underline how the meaning of physical therapy changes throughout the disease. Physical therapy in ALS has to be regarded as a supportive and palliative health care intervention beyond functional outcome parameters.

## Introduction

Amyotrophic lateral sclerosis (ALS) is a fatal neurodegenerative disease. The disease is characterized by a loss of motor neurons in the cortex, brain stem, and spinal cord resulting in progressive motor deficits and paralysis of the muscles that control limb movement, swallowing, and breathing [1]. As the disease progresses, muscles responsible for fine and gross motor functions are affected, leading to a decline in motor skills. As there is no current curative treatment for ALS, managing these complex symptoms depends on multidisciplinary care. Symptomatic, rehabilitative, and palliative therapy are typically delivered by a multiprofessional team that consists of neurologists, nurses, and therapists working in a coordinated and organized manner [2]. An important part of this multidisciplinary treatment is physical therapy, which is widely prescribed and applied in the treatment of ALS. A European survey has shown that 83% of ALS patients receive physical therapy [3]. Physiotherapists play an essential role on the multidisciplinary care team as they emphasize improving the function and quality of life in patients who require physical and functional dimensions of palliative care [4].

Experimental data [5-7] and several randomized clinical trials showed moderate effects and benefits of submaximal resistive exercises, especially in the early stages of the disease [8,9]. The neuromuscular mechanism was thought to prevent disuse atrophy and more efficient motor unit recruitment. Excessive or high resistance exercises have been associated with overwork damage and thus are not recommended in ALS treatment [8]. The key focus for physical therapists is to delay the decline of muscular strength by submaximal resistance exercise, which has been shown to be safe and efficacious. Additionally, because pain and spasticity worsen the burden of ALS, physical therapy also addresses these symptoms. Along with other service providers, physical therapists support the provision and adjustment of adaptive equipment and mobility aids [10]. However, there is still uncertainty about best practices concerning the manner, duration, and frequency of physical therapy. This lack of defined treatment guidelines arises from the large clinical heterogeneity of ALS syndromes, the different therapeutic approaches, and the individual expectations of patients and therapists.

Thus, this study aimed to:

1. Evaluate the frequency and duration of physical therapy sessions among ALS patients
2. Determine the most bothersome motor symptoms
3. Identify recommendation levels for physical therapy and the Net Promoter Score (NPS) at the beginning and end of the study

We investigated the recommendation of physical therapy to symptom progression in ALS. Furthermore, we explored whether the recommendation of physical therapy is related to the most concerning motor symptom, disease severity, duration, or the frequency of physical therapy sessions for ALS patients.

## Methods

### Study Design and Recruitment

This was a prospective, longitudinal, observational study that recruited a consecutive cohort of participants from the ALS outpatient department at Charité-Universitätsmedizin Berlin, Germany. A baseline assessment of epidemiological data, symptoms, type and amount of physical therapy was performed with 45 individuals, 20 of whom completed online surveys over a 6-months period, tracking symptom severity, restriction of activity, and recommendation for physical therapy.

### Setting

The digital and internet-supported case management network Ambulanzpartner Soziotechnologie (APST) was used for online self-assessment and evaluation of physical therapy [11]. APST encompasses the services of case management coordinators, a tailored digital management platform and assessment tools for self-evaluation, services, therapy and assistive devices [12]. Patients and their caregivers were granted access to the APST platform through personalized accounts.

### Participants

Inclusion criteria for this study involved a possible, probable or definitive diagnosis of ALS following the revised El Escorial criteria [13], a stage of a disease where at least one motor function was restricted, and participation in physical therapy. Patients with other severe life-limiting diseases or who showed clinically significant cognitive impairment were not eligible for this trial. For online assessment, participants used the digital case management program provided by APST [11].

### Variables and Data Sources

#### Physical Therapy

Physical therapy was prescribed by a neurologist specializing in ALS and undertaken by physical therapists trained in the treatment of neurological disorders including ALS. In addition to physical therapy, patients received special treatments such as massages, lymphatic drainage, thermal treatment, and breathing therapy if needed. The overall time and frequency of individual physical therapy sessions per week were documented, as were additional special treatments.

#### Amyotrophic Lateral Sclerosis Functional Rating Scale-Revised

We evaluated the functional impairment of participants using the ALS Functional Rating Scale-Revised (ALSFRS-R), through online self-assessment [14]. This scale is a validated and widely used instrument that gauges the fine and gross motor functions of the arms and legs, bulbar functions, and breathing abilities. It comprises 12 short, clear questions with 5 anchor points (0-4) for response options. Hence, the total range of the scale spans 0 to 48 points, with fewer points representing poorer functioning and higher disease severity. The loss of ALSFRS-R value per month, or delta ALSFRS-R, indicates the rate of deterioration and predicts survival [15].

#### Measure Yourself Medical Outcome Profile

To focus on specific bothersome or disabling motor symptoms we employed the Measure Yourself Medical Outcome Profile (MYMOP) [16,17]. This instrument has not been used in patients with ALS before but has been suggested as an individualized patient-reported outcome measure in primary care physical therapy [18]. The MYMOP is a brief, patient-generated, problem-specific questionnaire, which requires participants to specify a symptom that concerns them most. Subsequently, participants evaluate the severity of this symptom on a 7-point Likert scale (eg, weakness of the right leg could score 0 for “as good as it could be” to 6 for “as bad as it could be”) as compared to the previous week. The second part of the survey uses the same scale to assess whether the symptom is limiting or preventing a daily activity or movement, such as walking. Participants also rate general well-being. Follow-up questionnaires address the original concerns. All domains (symptom severity, restriction of activity, and well-being) can be analyzed individually or as a total score, the profile score, that equals the mean of the subscores recorded (score 0-6).

#### Net Promoter Score

**Figure 1:**
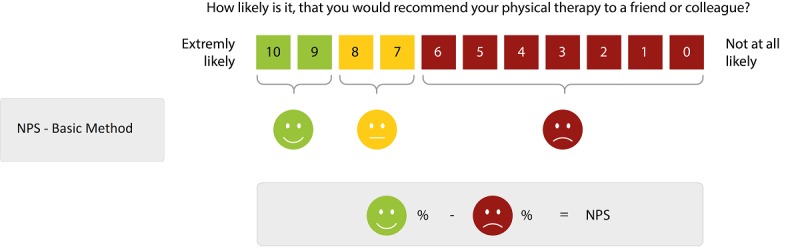
Net Promoter Score calculation method: promoters (green), indifferent (yellow), and detractors (red).

To evaluate the overall recommendation of physical therapy, we used a numeric rating scale (NRS) that derives from the Net Promoter Score (NPS) [19,20], which is used in customer relation management and has recently been introduced to clinical assessment [21,22]. The NPS is an easy-to-use, one-item questionnaire that is based on the question “How likely is it, that you would recommend the service to a friend or colleague?” Participants were asked to score on a 0 to 10 NRS, with 10 being extremely likely to recommend the therapy. The percentage of participants whose response was between 0-6 was subtracted from the percentage of those whose scores were 9-10 (Figure 1) to calculate the NPS. Participants with the values 7 and 8 were assumed to be indifferent or passive. Therefore, the NPS can be as low as –100 if everybody is a detractor, or as high as +100 if everybody is a promoter. A positive NPS is regarded as good, and an NPS of more than 50 is considered excellent. Alternatively, to avoid problems of NPS categorization, it is possible to refrain from calculating the NPS and only report the average NRS which reflects the recommendation [20].

### Data Analysis

Data were analyzed with IBM SPSS Statistics (Version 24.0) for Microsoft Windows. Results were expressed as mean (SD) if normally distributed and medians (maximum/minimum) if the distribution was non-Gaussian. Correlational analysis was performed with the Spearman rho (ρ) because of the ordinal nature of the scales. A statistically significant difference of paired samples was analyzed with a *t* test. The recommendation was tested with the Wilcoxon test for related samples. A *P* value of <.05 (two-tailed) was considered significant. Due to the observational design of the study, the data has not been adjusted for multiple comparisons.

### Protocol Approvals and Registrations

The study protocol was approved by the Medical Ethics Committee of the Charité-Universitätsmedizin Berlin, Germany. A data safety and monitoring board supervised the study. Signed patient information and informed consent forms were obtained from all participating patients.

## Results

### Descriptive Data

**Figure 2:**
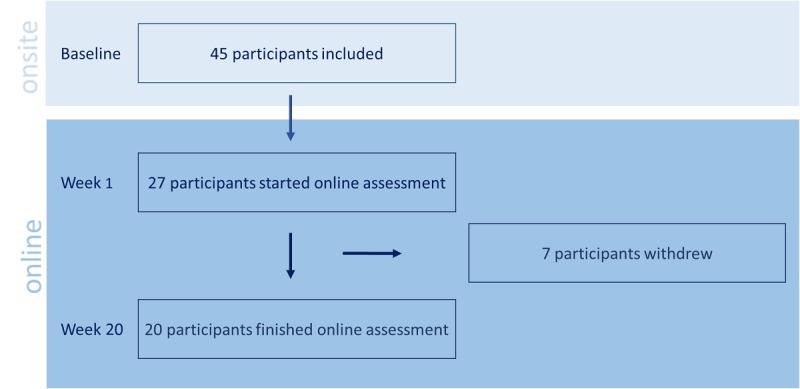
Flowchart of participants.

Forty-five participants were included in this study and performed the baseline assessment. Sixty percent (27/45) also consented to online assessment through MYMOP and the recommendation of physical therapy for 20 weeks. Twenty of the 45 (44%) participants finished the 20-week online assessment providing complete data sets (Figure 2).

The mean age of all participants at baseline was 59.2 years (SD 10.6) with a relatively long disease duration of 27 months (median min/max 3/203) due to a higher percentage of long-term survivors in our trial. The mean duration of physical therapy was 142.7 minutes per week (SD 60.4) and mean frequency was 2.9 sessions per week (SD 1.2). Occupational therapy and speech and language therapy are not included in these values. The demographics and baseline characteristics of the participants are presented in Table 1.

**Table 1:** Baseline characteristics of participants.

Characteristic		All patients, (N=45)		Online patients, (n=27)		Online patients-dropouts, (n=7)		Online patients-no dropouts, (n=20)	
Age (years), mean (SD)		59.2 (10.6)		59.4 (11.1)		58.0 (5.6)		59.9 (12.5)	
**Gender, n (%)**									
	Female		16 (36)		7 (35)		3 (43)		4 (20)
	Male		29 (64)		20 (65)		4 (57)		16 (80)
ALSFRS-R^a^ baseline, mean (SD)		36.9 (6.9)		38.5 (4.8)		36.1 (5.0)		39.4 (1.0)	
Delta ALSFRS-R^b^, mean (SD)		0.57 (0.51)		0.61 (0.57)		0.86 (0.27)		0.53 (0.11)	
Disease duration (months), median (min/max)		27.0 (3/203)		25.0 (3/194)		16.0 (9/87)		26.5 (3/195)	
Total MYMOP^c^ baseline, mean (SD)		3.0 (0.9)		3.0 (0.9)		3.1 (1.1)		2.9 (0.9)	
PT^d^ time per week (minutes), mean (SD)		142.7 (60.4)		151.1 (63.5)		157.1 (70.4)		149.0 (62.7)	
Overall time prescribed (minutes), mean (SD)		269.3 (138.6)		263.6 (110.7)		216.6 (58.5)		280.1 (120.7)	
PT frequency per week, mean (SD)		2.9 (1.2)		2.9 (1.2)		2.9 (1.5)		3.0 (1.2)	

^a^ALSFRS-R: Amyotrophic Lateral Sclerosis Functional Rating Scale-Revised.

^b^Loss of ALSFRS-R points per month.

^c^MYMOP: Measure Yourself Medical Outcome Profile.

^d^PT: physical therapy.

#### The Duration and Frequency of Physical Therapy

There was no significant difference between prescribed physical therapy time and session frequency in the different baseline cohorts. Given the fact that a regular physical therapy unit lasts between 45 to 60 minutes and patients receive 3 units per week, the mean duration of therapy sessions amounts to 2 and a half to 3 hours per week. Interestingly, an additional 2 hours per week were granted for special treatments. Only online participants who completed the study prematurely received fewer special treatment time of just 1 hour per week. However, this did not reach statistical significance (*P*=.19).

#### Disease Progression and Functional Impairment

The ALSFRS-R at baseline was comparable with other trials, but the ALS progression rate was 0.57 (SD 0.4), which is lower than in an average ALS population where the loss is usually 0.8 to 0.9 of a point [23]. In the online cohort, the constant decline in motor function was represented by an expected significant decline in the total ALSFRS-R from 39.4 to 36.4 (*P*=.05).

The 18/45 (40%) patients who did not participate in the online assessment showed a significantly more advanced stage of the disease compared to those who attended (ALSFRS-R: 34.4 versus 38.5, *P*=.05). Seven of 45 (16%) participants withdrew from the online survey after 7.7 weeks (SD 5.8). Among these participants a higher proportion was female (43% versus 20%) and tended to be more affected (ALSFRS-R: 36.1 versus 39.4, *P*=.13) with a higher rate of progression (delta ALSFRS-R: 0.72 versus 0.53, *P*=.19).

#### The Most Bothersome Symptom, Activity, and Well-Being

**Figure 3:**
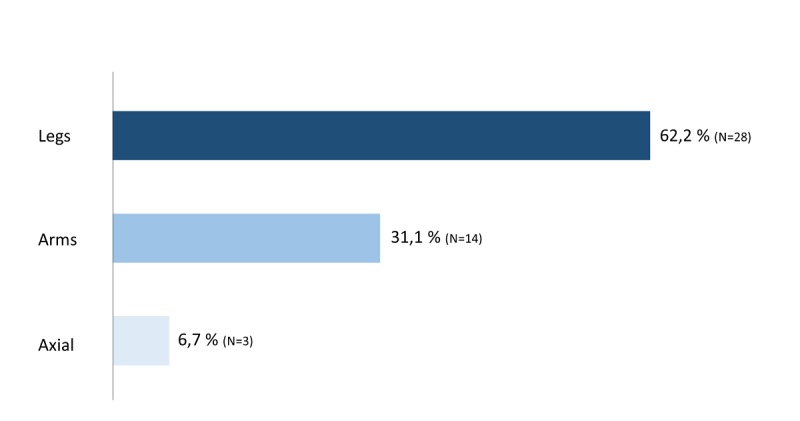
Distribution of the most concerning symptom at baseline (N=45).

Based on the initial MYMOP questionnaire, 62% (28/45) of participants defined symptoms in the legs as most bothersome, while 31% (14/45) cited restrictions in their arms as the most important issue. Three of the 45 participants (7%) named axial symptoms like torso weakness as the dominating symptom (Figure 3).

The total MYMOP of all cohorts at baseline was similar between 2.9 and 3.1. The profile score of MYMOP at baseline of 3.0 (SD 0.9, N=45) did not significantly correlate with the total ALSFRS-R at baseline (*r*=.27, *P*=.17). Whereas the correlation of the MYMOP with the according ALSFRS-R subscore related to functional loss of arms and legs was significant (*r*=.45, *P*=.003). However, the highest correlation was seen between the ALSFRS-R lower extremities subscore and the MYMOP symptom assessment subscore (*r*=.62, *P*<.001). This correlation was reproducible throughout the trial.

In the online cohort (n=20, Table 2) the profile score of MYMOP increased from 2.9 to 3.7 (*P*=.005). The MYMOP subscores for activity increased from 3.1 to 4.0 (*P*=.02). The burden of the target symptom increased from 3.1 to 3.9 (*P*=.02). The well-being subscore displayed a strong trend towards poorer well-being after 20 weeks (from 2.6 to 3.2) but without statistical significance (*P*=.08).

The 7/27 (26%) online participants who withdrew initially showed a poorer well-being subscore in the MYMOP as compared to participants who finished the assessment (3.4 versus 2.6, *P*=.08).

**Table 2:** Change over 20 weeks in the online cohort (n=20).

Outcome parameter	Baseline, mean (SD)	Week 20, mean (SD)	*P* value^a^
ALSFRS-R^b^	39.4 (1.0)	36.4 (1.3)	*<*.001
MYMOP^c^, profile	2.9 (0.2)	3.7 (0.2)	.005
MYMOP, well-being	2.6 (0.3)	3.2 (0.3)	.08
MYMOP, activity	3.1 (0.2)	4.0 (0.3)	.02
MYMOP, symptom	3.1 (0.2)	3.9 (0.3)	.02
Recommendation	7.6 (0.4)	8.6 (0.3)	.02
NPS^d^	20	50	—

^a^Wilcoxon test for related samples.

^b^ALSFRS-R: Amyotrophic Lateral Sclerosis Functional Rating Scale-Revised.

^c^MYMOP: Measure Yourself Medical Outcome Profile.

^d^NPS: Net Promotor Score.

#### Recommendation Levels for Physical Therapy

**Figure 4:**
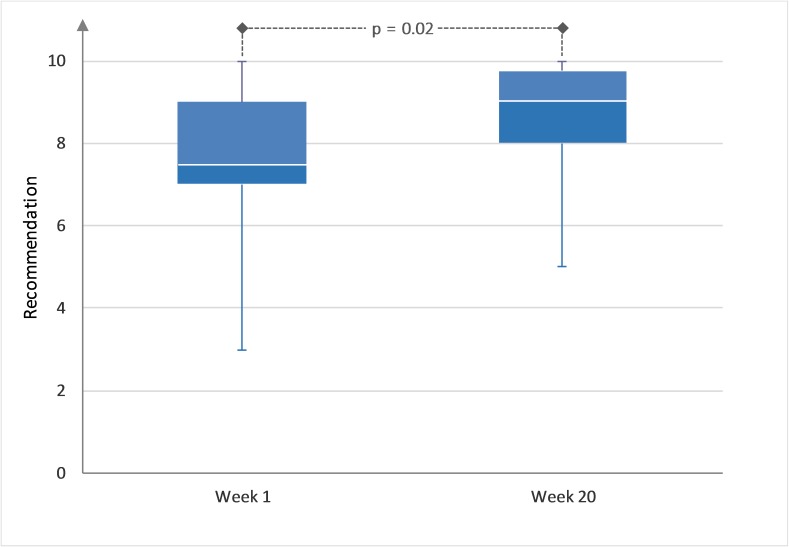
Recommendation of physical therapy at week 1 and at week 20 (*P*<.05).

The total value of the recommendation of physical therapy went from 7.6 to 8.6 (*P*=.02, Figure 4). In the 7/45 (16%) withdrawing participants, we could see a not statistically significant decrease of recommendation based on the last assessment before withdrawal: 7.4 (SD 2.2) versus 7.0 (SD 3.5).

**Figure 5:**
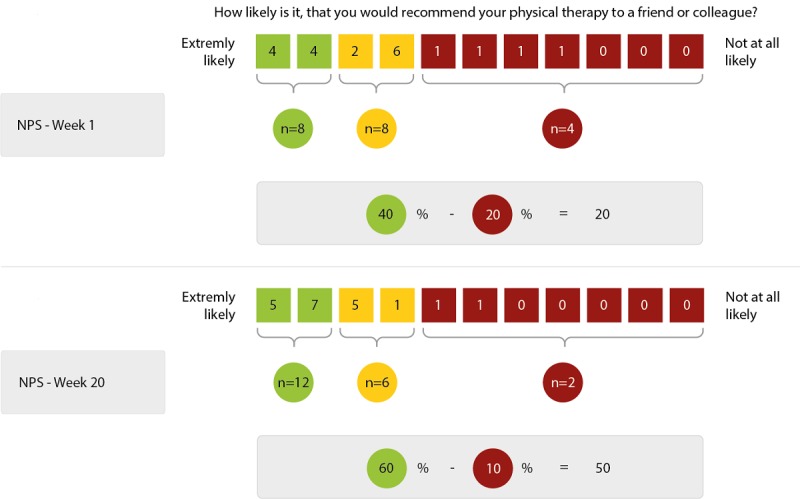
Change in Net Promoter Score from week 1 to week 20.

The recommendation was not influenced by the factors (1) age, (2) gender, (3) amount of physical therapy, (4) location of the most concerning symptom, (5) degree of functional impairment, and (6) well-being or activity (data not shown). Based on the recommendation we calculated the NPS, which increased from 20 at the beginning to 50 at the end of the observation interval (Figure 5).

## Discussion

### Principal Findings

This study aimed to determine patients’ perception of physical therapy during disease progression. To our knowledge, there are few systematic reports about the extent to which physical therapy is applied to ALS patients. Baseline assessment of this study revealed a mean duration of 269.3 minutes of prescribed physical therapy including special treatments and a mean frequency of three units per week. Our data did not show a significant correlation between the recommendation of physical therapy and the extent (duration and frequency) of its application.

The ALS progression, measured by the ALSFRS-R, was complimented by the MYMOP in order to show the effect of motor decline on the perception of physical therapy. The correlation between MYMOP and the motor domain of the ALSFRS-R was strong, although this score is unable to measure new or coexisting problems and concerns. The patient-centered assessment was unable to measure other perceived benefits, like the social and psychological meanings of physical therapy.

However, the studies found no evidence that declines in well-being, motor function, or levels of activity significantly degrade the overall recommendation of physical therapy. Remarkably, throughout the study, the rating of physical therapy improved in the majority of participants despite the functional decline. As shown by the NPS rating of 20 in the first online assessment, we found strong satisfaction with physical therapy. By week 20 the NPS value reached a value of 50, which is considered to be excellent and shows a high acceptance of physical therapy within the studied cohort.

### Limitations

Our findings must be considered in the context of their limitations. Out of all 45 participants, male patients were overrepresented in comparison to the general ALS population. This inadequacy was exacerbated in the online cohort, which we also have observed in previous online assessment trials. To reduce this bias, we attempted to recruit participants offline. However, women were more likely to terminate the assessment early. The 7 participants who discontinued online-assessment showed a trend towards faster progression, lower well-being and lower recommendation of physical therapy. Presumably, more aggressive disease progression might be a reason for dropping out, as might discontent with physical therapy. Measuring satisfaction using online self-assessment can be challenging. The NPS enables patients to rate physical therapy from the perspective of their own experience. At the same time, it is a 1-dimensional questionnaire and therefore assumed to be less reliable and more volatile than a composite index. In future studies, multidimensional or open designs should be considered to explore patients’ perspectives towards physical therapy in greater depth.

Further limitations in the study were the single center recruitment and the small sample size. Therefore, generalizations must be made with caution. Our cohort was representative of the ALS population regarding mean age and ALSFRS-R, but the participants showed a longer mean disease duration. The progression rate of 0.52 (SD 0.4) is lower than in an average ALS population, which is because a wider range of disease progression was represented as compared to homogenized populations within pharmaceutical trials. We can imagine that long-time survivors and patients whose diseases are progressing slowly will have a certain and eventually more positive attitude towards physical therapy, even though the effect of therapy on motor function might be considered more relevant in the early stages of the disease. Finally, our population was seen at a specialist center supported by a case management platform. Furthermore, it is located in an advanced country with a universal multi-payer health system where costs for physical therapy are covered mostly by compulsory health insurances. Consequently, our findings may not be broadly applicable to other populations.

### Conclusion

The overall positive assessment of physical therapy cannot be fully explained with the established rehabilitative concept of physical therapy. Our data suggest physical therapy plays an important role in a palliative context, where therapy and presumably the therapist hold considerable meaning for the patient. Physical therapists serving as interdisciplinary team members in palliative settings provide care for patients that extend beyond physical and bodily aims. Embracing this concept could entail shifting priorities across a disease continuum, and changing the perception of physical therapists as well as other allied health specialists [24]. Palliative and multidisciplinary approaches should be encouraged during the education, training, and qualification of physical therapists to implement the changing perceptions of physical therapy.

## Abbreviations

ALS: amyotrophic lateral sclerosis
ALSFRS-R: Amyotrophic Lateral Sclerosis Functional Rating Scale-Revised
APST: Ambulanzpartner Soziotechnologie APST GmbH
MYMOP: Measure Yourself Medical Outcome Profile
NPS: Net Promoter Score
NRS: numeric rating scale
PT: physical therapy
